# Using Restricted Cubic Splines to Study the Duration of Antibiotic Use in the Prognosis of Ventilator-Associated Pneumonia

**DOI:** 10.3389/fphar.2022.898630

**Published:** 2022-04-29

**Authors:** Yixian Xu, Didi Han, Fengshuo Xu, Si Shen, Xinkai Zheng, Hao Wang, Jun Lyu

**Affiliations:** ^1^ Department of Clinical Research, The First Affiliated Hospital of Jinan University, Guangzhou, China; ^2^ Department of Anesthesiology, The First Affiliated Hospital of Jinan University, Guangzhou, China; ^3^ School of Public Health, Xi’an Jiaotong University Health Science Center, Xi’an, China; ^4^ Medical Imaging Center, The First Affiliated Hospital of Jinan University, Guangzhou, China; ^5^ Department of Dermatology, The First Affiliated Hospital of Jinan University, Guangzhou, China; ^6^ Guangdong Provincial Key Laboratory of Traditional Chinese Medicine Informatization, Guangzhou, China

**Keywords:** MIMIC-IV1, ventilator-associated pneumonia, restricted cubic splines, duration of antibiotic use, intensive care unit, mortality, prediction

## Abstract

**Background:** Ventilator-associated pneumonia (VAP) is the most widespread and life-threatening nosocomial infection in intensive care units (ICUs). The duration of antibiotic use is a good predictor of prognosis in patients with VAP, but the ideal duration of antibiotic therapy for VAP in critically ill patients has not been confirmed. Research is therefore needed into the optimal duration of antibiotic use and its impact on VAP.

**Methods:** The Medical Information Mart for Intensive Care database included 1,609 patients with VAP. Chi-square or Student’s t-tests were used to compare groups, and Cox regression analysis was used to investigate the factors influencing the prognoses of patients with VAP. Nonlinear tests were performed on antibiotic use lasting <7, 7–10, and >10 days. Significant factors were included in the model for sensitivity analysis. For the subgroup analyses, the body mass indexes (BMIs) of patients were separated into BMI <30 kg/m2 and BMI ≥30 kg/m2, with the criterion of statistical significance set at *p* < 0.05. Restricted cubic splines were used to analyze the relationship between antibiotic use duration and mortality risk in patients with VAP.

**Results:** In patients with VAP, the effects of antibiotic use duration on the outcomes were nonlinear. Antibiotic use for 7–10 days in models 1–3 increased the risk of antibiotic use by 2.6020-, 2.1642-, and 2.3263-fold relative to for >10 days, respectively. The risks in models 1–3 for <7 days were 2.6510-, 1.9933-, and 2.5151-fold higher than those in models with >10 days of antibiotic use, respectively. These results were robust across the analyses.

**Conclusions:** The duration of antibiotic treatment had a nonlinear effect on the prognosis of patients with VAP. Antibiotic use durations of <7 days and 7–10 days both presented risks, and the appropriate duration of antibiotic use can ensure the good prognosis of patients with VAP.

## Introduction

Ventilator-associated pneumonia (VAP) is one of the most prominent hospital-acquired diseases in intensive care units (ICUs), and has a substantial risk of adverse effects (Xu et al., 2021). The prevalence of VAP is decreasing according to a hospital report from the United States Healthcare Safety Network, with about 10% of patients who require mechanical ventilation being diagnosed with VAP, a rate that has remained steady over the last decade. Each patient incurs additional VAP-related costs of approximately US$ 40,000 (Kalil et al., 2016). In the United States, epidemiological studies have confirmed that the incidence of VAP ranges from 2 to 16 per 1,000 ventilator days (Torres et al., 2017). VAP is recognized as pneumonia that occurs at least 48 h following endotracheal intubation or mechanical ventilation and is marked by infection-related symptoms such as widespread fever, new or progressive pulmonary infiltrations, and changes in the white blood cell count (Grief and Loza, 2018; Galhardo et al., 2020). VAP is associated with difficulty in weaning, prolonged mechanical ventilation and hospitalization times, and increased hospitalization costs for patients, which increase the economic costs to the health care system and consume huge medical resources (Wu et al., 2019; Pozuelo-Carrascosa et al., 2020).

Antibiotics are widely used to treat infectious diseases, and China is one of the world’s major producers and consumers of antibiotics. Antibiotics are used by about half of all hospital outpatients in China, according to linked publications, and antibiotic prescriptions account for about half of all medications dispensed by hospitals. The top five antibiotics taken by Chinese in 2013 were cephalexin, amoxicillin, ofloxacin, tetracycline, and norfloxacin (Qiao et al., 2018). Antibiotic resistance is higher in China than in Western countries, and drug-resistant microorganisms are becoming more common (Ding et al., 2019).

Previous reports suggest that infection causes one-third to one-half of all VAP-related deaths, with more fatalities for *Pseudomonas aeruginosa* and *Acinetobacter* species (Garnacho-Montero et al., 2005; Micek et al., 2015; Lima et al., 2020). Antibiotic resistance has also increased in common associated pathogens, and VAP risk is time-dependent, potentially leading to significant time-dependent biases, making it more difficult to empirically determine the duration of antibiotic use for these infections (Torres et al., 2017; Fernando et al., 2020; Bassetti et al., 2022). Pathogen diagnosis in patients with VAP typically requires an invasive quantitative culture of the lower respiratory tract using endotracheal aspirates, bronchoalveolar lavage fluid, protective specimen brushes, or semiquantitative noninvasive breath sampling (Arthur et al., 2016). However, due to the stringent growth requirements for detecting pathogens or other aspects of culture methods, important pathogens might not be detected, and it may be difficult to distinguish detected microorganisms from colonizing bacteria and actual pathogens.

While treatment is empirical until definitive results are available, the emergence of multidrug-resistant organisms, particularly in ICUs, necessitates determining the optimal antibiotic use duration (Timsit et al., 2017; Cilloniz et al., 2020; Yoo et al., 2020). In ICU patients, VAP is also the leading cause of antibiotic use (Koulenti et al., 2017), and guidelines recommend that adequate doses should be prescribed to patients with VAP to ensure early, appropriate, broad-spectrum antibiotic treatment, and to optimize the antibacterial treatment effect on patients with VAP by starting at the correct time. Appropriate and adequate treatment has been a consistent factor related to mortality rates (Guidelines for the management of, 2005). However, the importance of immediately administering antibiotics must always be matched against unnecessary hazards from antibiotics such as resistance and secondary infections (Lu et al., 2011).

Obese patients [body mass index (BMI) ≥30 kg/m2] had significantly lower 90-days mortality than nonobese patients (BMI <30 kg/m2) in a 2021 study (Nseir et al., 2021). BMI was also linked to a higher incidence of ICU-acquired infections. In obese patients, continuous antibiotics infusion can optimize the duration of time-dependent antibiotic exposure above the minimum inhibitory concentration (MIC), maximizing apoptosis (De Pascale et al., 2015). In patients with VAP, the optimal duration of antibiotic use could be used as a predictor for swift clinical assessments and prognostic research. It will be important to arrange innovative treatment strategies for patients with VAP if this indicator could effectively predict the probability of negative outcomes. Therefore, a more-thorough and precise insight into the role of an appropriate duration of antibiotic use in the prognosis of patients with VAP is critical.

The purpose of this study was to explore the effect of appropriate timing of antibiotic administration on the survival of VAP patients by applying restricted cubic splines (RCSs) to data in the Medical Information Market for Intensive Care (MIMIC) database.

## Materials and Methods

### Database

The Computational Physiology Laboratory of Massachusetts Institute of Technology, Beth Israel Deacon Medical Center, and Philips Healthcare jointly published the MIMIC-IV database (version 1.0) for the period of 2008–2019.

We received permission from an institutional review board of the Massachusetts Institute of Technology and Beth Israel Deaconess Medical Center for a longitudinal single-center database of clinical information on ICU patients, and data extraction from and access to the database were implemented (Yang et al., 2020). Dr. Yixian Xu, who completed the National Institutes of Health’s online training course, extracted the data (certification number: 39194349).

### Patients and Variables

**FIGURE 1:**
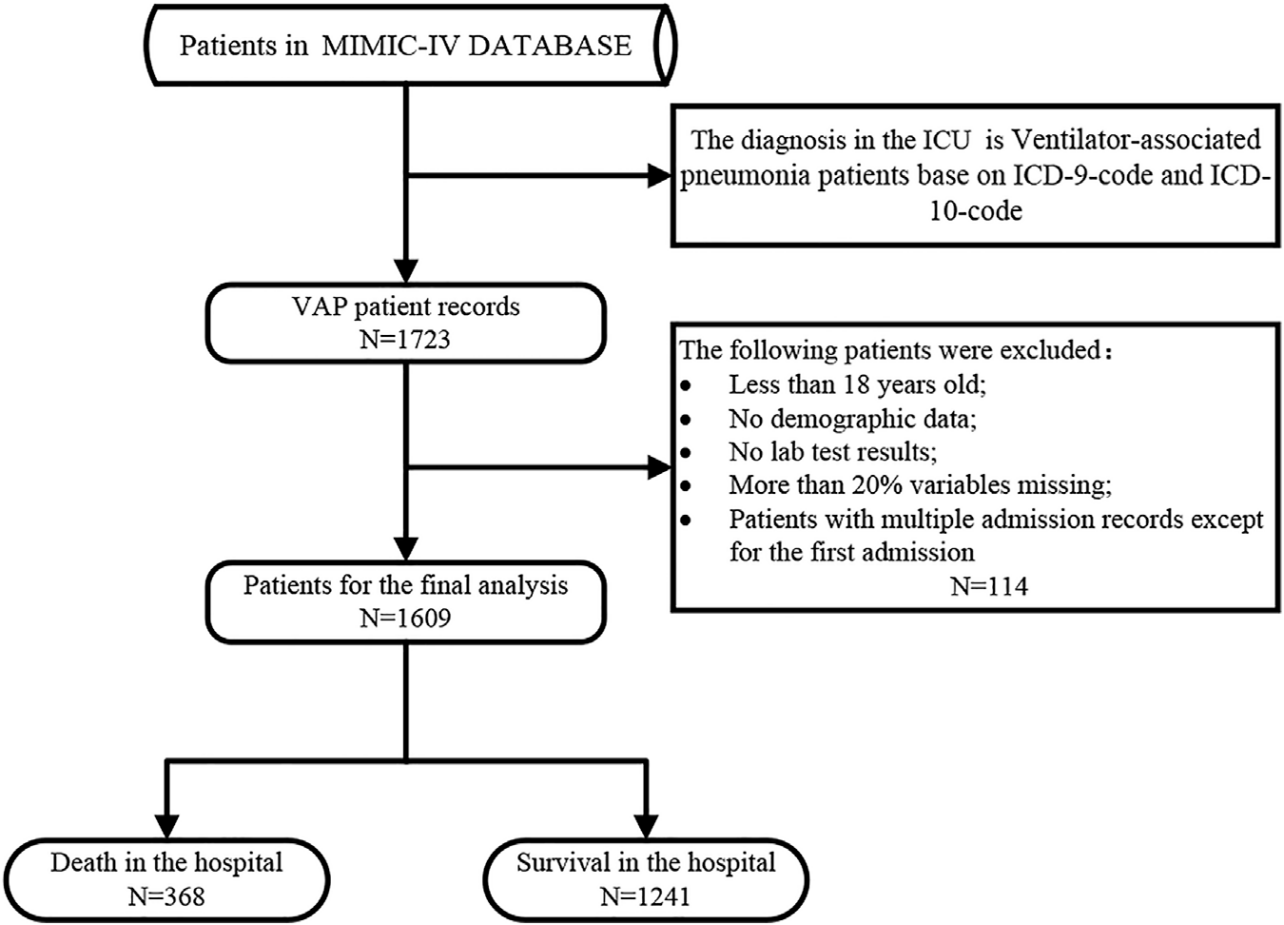
Flow chat of study selection.

Patients with VAP were identified in the MIMIC-IV database. This study gathered data on the demographics, laboratory tests, medications, vital signs, surgical procedures, and other personal information of patients with VAP (Wu et al., 2021). We extracted the data of 1,609 patients with VAP from the MIMIC-IV database for inclusion in this study. All patients were older than 18 years and had been admitted to the hospital for the first time. Figure 1 illustrates the exclusion and inclusion criteria of patients in this study.

The following variables were extracted from the records of the patients with VAP in the MIMIC-IV database: 1) Demographic data and vital signs at hospital admission [length of hospital stay, serum creatinine (SCR), antibiotic use days, urine output, mean blood pressure (MBP), diastolic blood pressure (DBP), admission age, temperature, and BMI]; 2) laboratory test data [red blood cell distribution width (RDW), hematocrit, hemoglobin, red blood cell (RBC), alanine aminotransferase (ALT), platelet count, bicarbonate, basophils, base excess, and total CO2]; 3) drugs and surgical operations [norepinephrine, vasopressin, cefazolin, meropenem, vancomycin oral liquid, tracheostomy, continuous invasive mechanical ventilation for less than 96 consecutive hours (CIMV), hemodialysis, gastric catheterization (GC), percutaneous (endoscopic) gastrostomy (PEG), performance of urinary filtration (POUF), respiratory ventilation for more than 96 consecutive hours (RV), temporary tracheostomy (TT), and venous catheterization for renal dialysis; and 4) complications and severity scoring systems [chronic kidney disease (CKD), Charlson Comorbidity Index (CCI), Sequential Organ Failure Assessment (SOFA), Acute Physiology Score-III (APSIII), Simplified Acute Physiology Score-II (SAPSII), Glasgow Coma Scale (GCS), Logistic Organ Dysfunction Score (LODS), and Model for End-stage Liver (MELD)]. The impact of antibiotic use duration on the survival of patients with VAP was investigated. The endpoint for this study was survival at discharge.

### Statistical Analysis

Patients were divided into two groups based on whether they survived during their hospitalization. Median and IQR values and mean ± SD values were used to summarize the nonnormally and normally distributed continuous variables, respectively. Student’s t-test, the Mann-Whitney *U* test, one-way ANOVA, and the Kruskal-Wallis H test were used to make comparisons of nonnormally distributed statistical data. The Kolmogorov-Smirnov test was used to determine if continuous variables were normally distributed. Categorical variables are expressed as percentages or numbers. We excluded confounding variables and outliers with considerable effects, and applied Chi-square tests to categorical variables and Student’s t-tests to continuous variables, with the exceptions of survival status and length of hospital stay.

Before performing the multivariate analysis, a univariate analysis was needed for each factor to determine if the covariance inclusion criteria were met to ascertain variables that affect VAP prognosis. Finally, the duration of antibiotic use was divided into >10, 7–10, and <7 days. A nonlinear test was used to determine whether the duration of antibiotics had a nonlinear effect on VAP prognosis. For subgroup analysis, the patients were divided into nonobese and obese groups.

Cox regression was performed, significant variables were added, three models were constructed, and the sensitivity models were tested. In the Cox regression analysis, model 1 only included the duration of antibiotic use, model 2 included some other variables, and model 3 included all key variables. Restricted cubic splines are good at dealing with the nonlinear relationship between continuous variables and response variables, and they can locate crucial key points. *p* < 0.05 was considered statistically significant. However, using data that had more than 20% missing or data that had missing points, we used the “vim” (Templ et al., 2012) and “MICE” (Zhang, 2016) (R software packages for multiple imputations using Templ’s method for exploration and data visualization. Excel and R (ggplot2, RMS package) were used for the statistical analysis.

## Results

### Patient Baseline Characteristics

Of the 1,609 patients with VAP, 368 died in the hospital. Variables from both groups were displayed and compared, and patients were separated into two groups depending on their survival status. Patients who survived at a median age of 63 (51, 73) days were younger than those who died at 70 (60, 80) years. Surviving patients were less likely to develop complications such as hypertension, chronic obstructive pulmonary disease (COPD), and diabetes than those who died. All variables affected VAP prognosis differently in this study. The baseline characteristics, laboratory parameters, and vital signs of survivors and deceased patients during hospitalization are listed in Table 1.

**TABLE 1:** Baseline characteristics of patients in the study.

Variable		Live	Dead	p-Value
N	—	1,241	368	<0.001
Los_hospital	—	23.00 [16.00, 33.00]	16.00 [10.00, 25.00]	
CKD	No	1,000 (80.6)	259 (70.4)	<0.001
	Yes	241 (19.4)	109 (29.6)	
Norepinephrine	No	659 (53.1)	133 (36.1)	<0.001
	Yes	582 (46.9)	235 (63.9)	
Vasopressin	No	1,021 (82.3)	252 (68.5)	<0.001
	Yes	220 (17.7)	116 (31.5)	
CefazoLIN	No	913 (73.6)	306 (83.2)	<0.001
	Yes	328 (26.4)	62 (16.8)	
Meropenem	No	982 (79.1)	271 (73.6)	0.031
	Yes	259 (20.9)	97 (26.4)	
Vancomycin oral liquid	No	1,122 (90.4)	316 (85.9)	0.017
	Yes	119 (9.6)	52 (14.1)	
Tracheostomy	No	1,105 (89.0)	344 (93.5)	0.016
	Yes	136 (11.0)	24 (6.5)	
CIMV	No	1,068 (86.1)	339 (92.1)	0.003
	Yes	173 (13.9)	29 (7.9)	
Hemodialysis	No	1,131 (91.1)	314 (85.3)	0.002
	Yes	110 (8.9)	54 (14.7)	
Gastric catheterization	No	1,074 (86.5)	345 (93.8)	<0.001
	Yes	167 (13.5)	23 (6.2)	
PEG	No	981 (79.0)	349 (94.8)	<0.001
	Yes	260 (21.0)	19 (5.2)	
POUF	No	1,203 (96.9)	330 (89.7)	<0.001
	Yes	38 (3.1)	38 (10.3)	
RV	No	838 (67.5)	217 (59.0)	0.003
	Yes	403 (32.5)	151 (41.0)	
Temporary tracheostomy	No	947 (76.3)	333 (90.5)	<0.001
	Yes	294 (23.7)	35 (9.5)	
Venous catheterization for renal dialysis	No	1,155 (93.1)	329 (89.4)	0.028
	Yes	86 (6.9)	39 (10.6)	
Antibiotics_day	—	13.00 [7.00, 20.00]	10.00 [5.75, 17.00]	<0.001
APSIII	—	64.00 [48.00, 84.00]	77.50 [59.00, 100.25]	<0.001
Baseexcess	—	0 [−4.00, 2.00]	−1.00 [−5.00, 1.00]	0.029
Totalco2	—	25.00 [22.00, 28.00]	24.00 [21.00, 28.00]	0.012
Charlson_comorbidity_index	—	5.00 [3.00, 7.00]	7.00 [5.00, 8.00]	<0.001
SCR	—	0.60 [0.40, 0.90]	0.80 [0.50, 1.10]	<0.001
GCS	—	9.00 [6.00, 13.00]	7.00 [3.00, 12.00]	<0.001
SOFA	—	8.00 [5.00, 11.00]	9.00 [6.00, 12.00]	<0.001
LODS	—	8.00 [5.00, 10.00]	9.00 [7.00, 12.00]	<0.001
MELD	—	10.00 [10.00, 20.00]	20.00 [10.00, 22.64]	<0.001
SAPSII	—	39.00 [30.00, 49.00]	46.50 [37.00, 55.00]	<0.001
BMI	—	28.44 [24.45, 34.41]	27.15 [23.17, 32.60]	0.001
Urineoutput	—	1,574.00 [915.00,2350.00]	1,146.00 [598.75,2025.00]	<0.001
DBP	—	62 [57, 69]	60 [53, 67]	0.001
MBP	—	78 [71, 86]	76 [69, 84]	0.004
Temperature	—	37.1 [36.8, 37.5]	36.9 [36.6, 37.3]	<0.001
Admission_age	—	63.00 [51.00, 73.00]	70.00 [60.00, 80.00]	<0.001
Hematocrit	—	33.90 [29.00, 39.00]	32.50 [27.90, 37.52]	0.011
Hemoglobin	—	11.20 [9.50, 13.10]	10.70 [9.00, 12.53]	0.004
RBC	—	3.71 [3.13, 4.31]	3.50 [2.99, 4.13]	<0.001
RDW	—	14.40 [13.40, 16.00]	15.00 [13.60, 16.80]	<0.001
ALT	—	1,356.00 [613.00,3024.00]	1,081.50 [516.00,2658.50]	0.031
Platelet_Count	—	886.00 [756.00, 1,024.00]	852.50 [725.00, 996.75]	0.027
Bicarbonate	—	42.00 [39.00, 45.00]	41.00 [39.00, 44.00]	0.010
Basophils	—	3.00 [2.00, 5.00]	3.00 [2.00, 4.00]	0.019

CKD, chronic kidney disease; CIMV, continuous invasive mechanical ventilation for less than 96 consecutive hours; PEG, percutaneous (endoscopic) gastrostomy; POUF, performance of urinary filtration; RV, respiratory ventilation for more than 96 consecutive hours; APSIII, Acute Physiology Score III; SCR, serum creatinine; GCS, glasgow coma scale; SOFA, sequential organ failure assessment; LODS, logistic organ dysfunction score; Meld, Model for End-stage Liver Disease; SAPSII, Simplified Acute Physiology Score II; BMI, body mass index; DBP, diastolic blood pressure; MBP, mean blood pressure; RBC, red blood cell; RDW, red blood cell distribution width; ALT, alanine aminotransferase.

### Restricted Cubic Splines in the Cox Proportional-Hazards Regression Model

In the univariate Cox regression analysis, model 3 included all variables for which *p* < 0.05. The results indicated that CKD, norepinephrine, vasopressin, cefazolin, tracheostomy, GC, PEG, POUF, TT, antibiotic use duration, APSIII, CCI, SCR, GCS score, SOFA score, LODS, MELD score, SAPSII, BMI, urine output, DBP, MBP, temperature, admission age, hemoglobin, RBC, RDW, ALT, platelet count, bicarbonate, and basophils were prognostic factors for patients with VAP (all *p* < 0.05, Table 2).

**TABLE 2:** The results of univariate cox regression analysis.

Variable	HR	95%CI	*p*-Value
Antibiotics_day	0.96	0.96–0.97	<0.001
CKD			
No	Reference	—	
Yes	1.43	1.14–1.79	0.002
Norepinephrine			
No	Reference	—	
Yes	1.45	1.18–1.80	0.001
Vasopressin			
No	Reference	—	
Yes	1.44	1.15–1.79	0.001
CefazoLIN			
No	Reference	—	
Yes	0.52	0.40–0.69	0.001
Tracheostomy			
No	Reference	—	
Yes	0.37	0.24–0.56	<0.001
Gastric catheterization			
No	Reference	—	
Yes	0.29	0.19–0.45	<0.001
PEG			
No	Reference	—	
Yes	0.20	0.13–0.32	<0.001
POUF			
No	Reference	—	
Yes	1.84	1.31–2.57	<0.001
Temporary tracheostomy			
No	Reference	—	
Yes	0.29	0.20–0.41	<0.001
APSIII	1.01	1.01–1.01	<0.001
Charlson_comorbidity_index	1.13	1.09–1.17	<0.001
SCR	1.38	1.24–1.54	<0.001
GCS	0.97	0.95–1.00	0.022
SOFA	1.04	1.01–1.06	0.003
LODS	1.08	1.05–1.11	<0.001
MELD	1.02	1.01–1.03	<0.001
SAPSII	1.02	1.01–1.03	<0.001
BMI	0.98	0.97–0.99	0.003
Urineoutput	1.00	1.00–1.00	<0.001
DBP	0.98	0.97–0.99	<0.001
MBP	0.98	0.97–0.99	0.001
Temperature	0.76	0.67–0.85	<0.001
Admission_age	1.03	1.03–1.04	<0.001
Hemoglobin	0.95	0.92–0.99	0.011
RBC	0.86	0.77–0.96	0.006
RDW	1.05	1.01–1.09	0.007
ALT	1.00	1.00–1.00	0.006
Platelet_Count	1.00	1.00–1.00	<0.001
Bicarbonate	0.93	0.91–0.95	<0.001
Basophils	0.95	0.91–0.98	0.001

CKD, chronic kidney disease; PEG, percutaneous (endoscopic) gastrostomy; POUF, performance of urinary filtration; RV, respiratory ventilation for more than 96 consecutive hours; APSIII, Acute Physiology Score III; SCR, serum creatinine; GCS, glasgow coma scale; SOFA, sequential organ failure assessment; LODS, logistic organ dysfunction score; Meld, Model for End-stage Liver Disease; SAPSII, Simplified Acute Physiology Score II; BMI, body mass index; DBP, diastolic blood pressure; MBP, mean blood pressure; RBC, red blood cell; RDW, red blood cell distribution width; ALT, alanine aminotransferase; CI, confidence interval; HR , hazard ratios.

**FIGURE 2:**
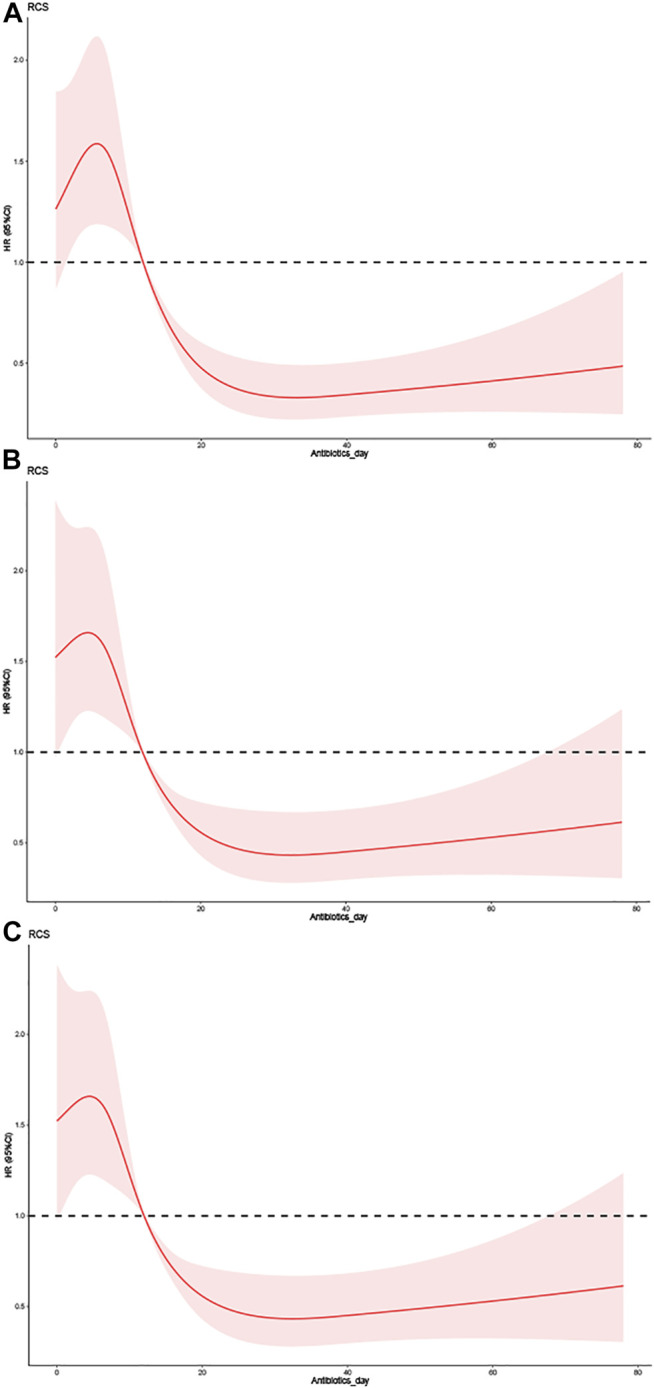
The effect of different doses of the duration of antibiotic use on the prognosis of VAP. **(A)** Univariate. **(B)** Adjusted for CKD, Norepinephrine, Vasopressin, CefazoLIN, Tracheostomy, GC, PEG, POUF, TT, SCR, BMI, Urineoutput, DBP, MBP, Antibiotics day, Temperature, Admission age, Hemoglobin, RBC, RDW, ALT, Platelet Count, Bicarbonate and Basophils. **(C)** Adjusted for Antibiotics day, CKD, Norepinephrine, Vasopressin, CefazoLIN, Tracheostomy, GC, PEG, POUF, TT, APSIII, Charlson comorbidity index, SCR, GCS, SOFA, LODS, MELD, SAPSII, BMI, Urineoutput, DBP, MBP, Temperature, Admission age, Hemoglobin, RBC, RDW, ALT, Platelet Count, Bicarbonate and Basophils.

RCSs were used to perform the association analysis between duration of antibiotic use and risk of all-cause mortality from VAP. The RCS test yielded statistically significant findings (Table 3). Model 1 included univariate (antibiotic use duration) analyses, as illustrated in Figure 2A. The hazard ratios (HRs) of model 1 were 2.6020 (95% confidence interval [CI] = 1.8940– 3.5750, *p* < 0.001) and 2.6510 (95% CI = 1.9880–3.5360, *p* < 0.01) for antibiotic use durations of 7–10 and <7 days, respectively. Model 2 included CKD, norepinephrine, vasopressin, cefazolin, tracheostomy, GC, PEG, POUF, TT, SCR, BMI, urine output, DBP, MBP, antibiotic use duration, temperature, admission age, hemoglobin, RBC, RDW, ALT, platelet count, bicarbonate, and basophils (Figure 2B). Model 2 had HRs of 2.1642 (95% CI = 1.5394–3.0426, *p* < 0.001) and 1.9933 (95% CI = 1.4077–2.8226, *p* < 0.001) for antibiotic use durations of 7–10 and <7 days, respectively. As demonstrated in Figure 2C, the significant variables from the Cox univariate regression analysis were incorporated into Model 3. Model 3 had HRs of 2.3263 (95% CI = 1.6375–3.3047, *p* < 0.001) and 2.5151 (95% CI = 1.7424–3.6307, *p* < 0.001) for antibiotic use durations of 7–10 and <7 days, respectively. In all models, the prognostic curves for VAP were nonlinear for antibiotic use duration. the antibiotic use duration curve showed a significant change trend at around 12 days (Figure 2, all *p* < 0.001). Notably, as the adjustment variable increased, the risk for antibiotic use for ≤10 days was more pronounced, and statistically significant that for >10 days.

**TABLE 3:** Cox regression analyses of the relationship between the duration of antibiotic use and VAP prognosis.

Variable	Model1	Model2	Model3
	HR (95%CI) *p*-value	HR (95%CI) *p*-value	HR (95%CI) *p*-value
>10 days	1	1	1
7–10 days	2.602 (1.894–3.575) <0.001	2.1642 (1.5394–3.0426) <0.001	2.3263 (1.6375–3.3047) <0.001
<7 days	2.651 (1.988–3.536) <0.001	1.9933 (1.4077–2.8226) <0.001	2.5151 (1.7424–3.6307) <0.001

Model 1: univariate.

Model 2: adjust for CKD, norepinephrine, vasopressin, cefazolin, tracheostomy, GC, PEG, POUF, TT, SCR, BMI, urine output, DBP, MBP, antibiotic use duration, temperature, admission age, hemoglobin, RBC, RDW, ALT, platelet count, bicarbonate, and basophils.

Model 3: adjusted forAntibiotics day, CKD, norepinephrine, Vasopressin, CefazoLIN, tracheostomy, GC, PEG, POUF, TT, APSIII, charlson comorbidity index, SCR, GCS, SOFA, LODS, MELD, SAPSII, BMI, urineoutput, DBP, MBP, temperature, Admission age, Hemoglobin, RBC, RDW, ALT, platelet count, Bicarbonate and Basophils.

### Subgroup Analysis of the Restricted Cubic Spline Cox Proportional-Hazards Regression Model

**FIGURE 3:**
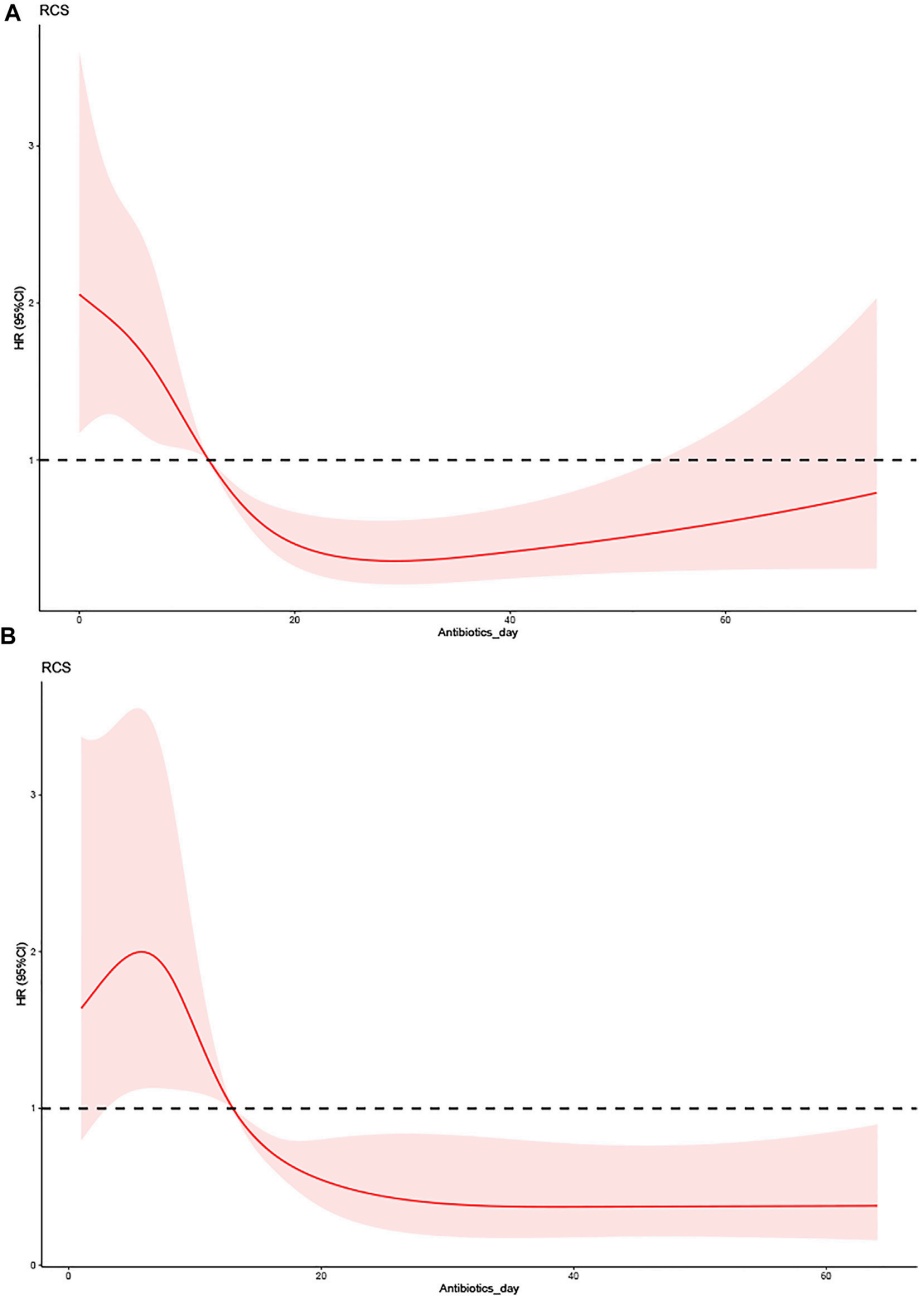
The effect of the duration of antibiotic use on the prognosis of VAP between different BMI groups. **(A)** Low-BMI group (BMI<30 kg/m2). **(B)** High-BMI group (BMI ≥30 kg/m2).

The Cox regression analysis revealed that BMI was a predictive factor for patients with VAP. Tables 4, 5 list the baseline characteristics of each subgroup and the Cox regression analysis findings, with the outcomes of both groups being nonlinear (*p* < 0.05). Figure 3 depicts the nonlinear curve comparison between the two groups. The curve of antibiotic use time was higher in the nonobese than the obese group. This result reflects that patients with VAP and low BMI have a higher risk from an antibiotic use duration >10 days than those with high BMI. These results were the same in all models.

**TABLE 4:** Baseline characteristics between different BMI groups.

Variable	—	BMI<30 kg/m2	BMI≥30 kg/m2	*p*-Value
N	—	948	661	0.009
Los_hospital	—	21 [14, 30]	22 [15, 33]	
Status	No	710 (74.9)	531 (80.3)	0.011
	Yes	238 (25.1)	130 (19.7)	
CKD	No	750 (79.1)	509 (77.0)	0.343
	Yes	198 (20.9)	152 (23.0)	
Norepinephrine	No	474 (50.0)	318 (48.1)	0.487
	Yes	474 (50.0)	343 (51.9)	
Vasopressin	No	764 (80.6)	509 (77.0)	0.093
	Yes	184 (19.4)	152 (23.0)	
CefazoLIN	No	711 (75.0)	508 (76.9)	0.427
	Yes	237 (25.0)	153 (23.1)	
Meropenem	No	747 (78.8)	506 (76.6)	0.314
	Yes	201 (21.2)	155 (23.4)	
Vancomycin oral liquid	No	854 (90.1)	584 (88.4)	0.304
	Yes	94 (9.9)	77 (11.6)	
Tracheostomy	No	856 (90.3)	593 (89.7)	0.764
	Yes	92 (9.7)	68 (10.3)	
CIMV	No	815 (86.0)	592 (89.6)	0.039
	Yes	133 (14.0)	69 (10.4)	
Hemodialysis	No	871 (91.9)	574 (86.8)	0.001
	Yes	77 (8.1)	87 (13.2)	
Gastric catheterization	No	830 (87.6)	589 (89.1)	0.383
	Yes	118 (12.4)	72 (10.9)	
PEG	No	766 (80.8)	564 (85.3)	0.022
	Yes	182 (19.2)	97 (14.7)	
POUF	No	915 (96.5)	618 (93.5)	0.007
	Yes	33 (3.5)	43 (6.5)	
RV	No	626 (66.0)	429 (64.9)	0.677
	Yes	322 (34.0)	232 (35.1)	
Temporary tracheostomy	No	760 (80.2)	520 (78.7)	0.502
	Yes	188 (19.8)	141 (21.3)	
Venous catheterization for renal dialysis	No	894 (94.3)	590 (89.3)	<0.001
	Yes	54 (5.7)	71 (10.7)	
Antibiotics_day	>10 days	540 (57.0)	387 (58.5)	0.155
	7–10 days	172 (18.1)	135 (20.4)	
	<7 days	236 (24.9)	139 (21.0)	
APSIII	—	66.00 [49.00, 86.00]	71.00 [52.00, 91.00]	0.011
Baseexcess	—	−1.00 [−5.00, 1.00]	0.00 [−4.00, 2.00]	0.061
Totalco2	—	24.00 [21.00, 28.00]	25.00 [22.00, 29.00]	0.001
Charlson_comorbidity_index	—	6.00 [4.00, 8.00]	6.00 [3.00, 8.00]	0.610
SCR	—	0.60 [0.40, 0.90]	0.70 [0.50, 1.00]	<0.001
GCS	—	8.00 [5.00, 12.00]	9.00 [4.00, 13.00]	0.909
SOFA	—	7.00 [5.00, 11.00]	8.00 [5.00, 12.00]	0.001
LODS	—	8.00 [5.00, 10.00]	8.00 [6.00, 11.00]	0.027
MELD	—	10.00 [10.00, 20.00]	10.00 [10.00, 21.32]	<0.001
SAPSII	—	41.00 [31.00, 51.00]	41.00 [31.00, 50.00]	0.988
Urineoutput	—	1,468.50 [859.50,2292.50]	1,500.00 [814.00,2325.00]	0.818
DBP	—	61 [56, 69]	62 [56, 69]	0.656
MBP	—	77 [71, 85]	78 [71, 86]	0.830
Temperature	—	37.1 [36.7, 37.5]	37.1 [36.8, 37.5]	0.011
Admission_age	—	67.00 [54.00, 78.00]	63.00 [52.00, 72.00]	<0.001
Hematocrit	—	32.70 [28.60, 37.90]	34.70 [29.10, 39.60]	<0.001
Hemoglobin	—	10.90 [9.30, 12.80]	11.30 [9.60, 13.20]	0.005
RBC	—	3.55 [3.04, 4.18]	3.82 [3.17, 4.42]	<0.001
RDW	—	14.40 [13.40, 16.00]	14.60 [13.50, 16.30]	0.014
ALT	—	1,236.50 [586.00,2922.00]	1,335.00 [598.00, 3,137.00]	0.503
Platelet_Count	—	880.00 [749.75,1031.00]	872.00 [755.00, 999.00]	0.384
Bicarbonate	—	41.00 [39.00, 45.00]	42.00 [39.00, 45.00]	0.255
Basophils	—	3.00 [2.00, 4.00]	3.00 [2.00, 4.20]	0.768

**TABLE 5:** univariate cox regression results for different BMI groups.

Variable	Low-BMI group (BMI<30 kg/m2)			High-BMI group (BMI≥30 kg/m2)		
	HR	95%CI	*p*-value	HR	95%CI	*p*-value
Antibiotics_day						
>10 days	Reference	—	—	—	—	—
7–10 days	2.53	1.80–3.54	<0.001	2.60	1.69–4.00	0.001
<7 days	2.74	2.04–3.68	<0.001	2.72	1.78–4.14	<0.001
CefazoLIN						
No	Reference	—	—	—	—	—
Yes	0.52	0.37–0.73	<0.001	0.52	0.32–0.83	0.006
Tracheostomy						
No	Reference	—	—	—	—	—
Yes	0.34	0.20–0.58	<0.001	0.41	0.21–0.81	0.010
Gastric catheterization						
No	Reference	—	—	—	—	—
Yes	0.31	0.19–0.50	<0.001	0.24	0.11–0.55	0.001
PEG						
No	Reference	—	—	—	—	—
Yes	0.16	0.09–0.28	<0.001	0.29	0.14–0.63	0.002
POUF						
No	Reference	—	—	—	—	—
Yes	2.02	1.26–3.24	0.004	1.93	1.18–3.14	0.009
Temporary tracheostomy						
No	Reference	—	—	—	—	—
Yes	0.26	0.16–0.41	<0.001	0.34	0.20–0.59	<0.001
APSIII	1.01	1.01–1.01	<0.001	1.01	1.00–1.01	0.005
Charlson_comorbidity_index	1.14	1.1–1.19	<0.001	1.11	1.05–1.18	<0.001
SCR	1.40	1.25–1.57	<0.001	1.38	1.1–1.71	0.005
SOFA	1.04	1.01–1.07	0.006	1.04	1.00–1.08	0.049
LODS	1.08	1.04–1.12	<0.001	1.09	1.03–1.14	0.001
MELD	1.02	1.00–1.03	0.018	1.04	1.02–1.06	<0.001
SAPSII	1.02	1.01–1.02	<0.001	1.03	1.01–1.04	<0.001
Urineoutput	1.00	1.00–1.00	0.003	1.00	1.00–1.00	0.019
Admission_age	1.03	1.02–1.04	<0.001	1.04	1.03–1.05	<0.001
Bicarbonate	0.93	0.91–0.96	<0.001	0.93	0.89–0.97	<0.001

## Discussion

VAP is a primary risk factor for mortality among critically ill patients. Treatment strategies may benefit from objective prognostic assessment tools (International Symposium on Intensive Care, 2016). Due to the obvious severity of VAP and the relative vulnerability of critically ill patients, timely and appropriate antimicrobial therapy is critical for reducing its burden, and infection control is implemented by avoiding prolonged ventilation with adequate sedation and weaning and avoiding endotracheal tubes. Biofilm formation, microaspiration of subglottic secretions, and bacterial translocation from the stomach to the upper respiratory tract and oropharynx are the basis for colonization (Martin-Loeches et al., 2018). Some studies have classified VAP into early-onset and late-onset types based on time of admission, but the bacteriological differences between the two are unclear. As an outcome, antibiotic selection based on pneumonia onset may result in over-and undertreatment of broad-spectrum drugs, so determining the perfect duration of antibiotic use for patients with VAP is critical for ensuring good clinical outcomes (Nair and Niederman, 2015; Torres et al., 2017).

Antibiotic stewardship methods in China include bacterial resistance surveillance at the hospital level, as local antibiotic resistance statistics are crucial for guiding rational antibiotic usage, and the development of antibiotic resistance is closely related to the duration of antibiotic use. In this research, a reliable predictive model for the duration of antibiotic treatment in patients with ventilator-associated pneumonia was developed, which can assist clinicians in making decisions about the prognosis of VAP patients and the optimal duration of antibiotic treatment, improving the patient’s chance of survival and reducing the risk of social labor loss cost and national medical resources (Hu et al., 2018; Chen et al., 2021).

Globally, overuse and inappropriate use of antibiotics is widely known as the primary cause of antibiotic resistance, as well as prolonging hospital stays, increasing treatment costs, and mortality associated with infectious diseases, acute upper respiratory infections, and ventilator-related complications. The most prevalent illnesses linked to antibiotic misuse are acute upper respiratory tract infection and ventilator-associated pneumonia. Antibiotic resistance has posed a serious threat to the effectiveness of existing antibiotics and their prescription regimens, affecting people of all socioeconomic backgrounds in community-acquired and nosocomial infections, as well as high health-care costs that lead to chronic poverty, whether untreated or treated. Excessive risk of morbidity and mortality. Appropriate antibiotic treatment length can effectively prevent drug resistance, better reduce human capital loss, mitigate economic shocks suffered by poor and developing societies and countries, and lower the risk of medical poverty traps (Ahmad and Khan, 2019; Zhao et al., 2020).

The bactericidal effect of antibiotics has been observed to depend on the time when the MIC is exceeded, and it is recommended to reach a time where the MIC is 100% or even higher. It is reasonable to apply a positive PharmacoKinetics and PharmacoDynamics (PK/PD) target in patients with VAP. In patients with respiratory tract infection, the 30-days survival rate of those receiving long-term β-lactam infusion was dramatically higher than that of patients receiving an intermittent bolus, and antibiotics had a considerable degree of concentration-dependent apoptosis characteristics as well as time-dependent characteristics (Timsit et al., 2017). The optimal duration of antibiotic use should therefore be invoked as a prognostic indicator to detect the clinical outcomes of patients with VAP.

A Cox proportional-hazards regression model fitted with RCS was used in this study to explore the link between the duration of antibiotic use and the risk of all-cause mortality. Compared with a duration of antibiotic use >10 days, patients with VAP and a duration of ≤10 days had a higher mortality risk. The Cox multivariate analysis indicated that the duration of antibiotic use was a prognostic factor for patients with VAP (*p* < 0.001). All prediction curves for antibiotic use time in RCS had similar performances. Based on antibiotic use of >10 days, this study set a long-term antibiotic range within which the risk of VAP was lowest. Notably, the effect of antibiotic use duration on VAP prognosis was nonlinear with statistical significance (*p* < 0.001). When performing sensitivity analyses, all models with antibiotic use ≤10 days also had a higher mortality risk than those with >10 days. For models 1-3, the risks of using antibiotics for 7–10 days were 2.6020-, 2.1642-, and 2.3263-fold higher than for an antibiotic use duration of >10 days, respectively, while the risks of model 1–3 with an antibiotic use duration of <7 days were 2.6020-, 2.1642-, and 2.3263-fold higher, respectively.

In the subgroup analyses, the nonobese and obese groups also had a higher risk from antibiotic use ≤10 days compared with >10 days. According to Pugh et al., the VAP recurrence rate was higher for a short course of 8 days than for a longer course of 12–15 days in patients with VAP from nonfermenting Gram-negative bacilli organisms. This increased recurrence risk did not appear to be linked to increased mortality, the need for prolonged mechanical ventilation, or the time spent in the ICU (Pugh et al., 2015). Insufficient empirical antibiotic treatment has been linked to increased mortality risk (Hanes et al., 2002), and Rhodes et al. found that patients with VAP are a changing population at risk of antibiotic resistance and an underdosing response to altered antibiotic pharmacokinetic characteristics (Rhodes et al., 2018). Initial empirical treatments for VAP should therefore use antibiotics to cover all possible pathogens, including *Acinetobacter baumannii*, *Pseudomonas aeruginosa*, *Stenotrophomonas maltophilia*, and *Methicillin-resistant Staphylococcus aureus*, and pathogen identification and drug susceptibility assessments should be carried out as soon as possible. Trials to ensure the adequate coverage and appropriate dose and duration of treatment could reduce the mortality from VAP (Wang et al., 2018; Gore et al., 2020).

De Oliveira noted that across all age groups, obese patients were not more likely to develop pulmonary complications than those with normal BMI (De Oliveira et al., 2017). Wardell et al. found that obesity was not necessarily associated with poor prognoses among critically ill patients (Wardell et al., 2015). This result was consistent with recent research that confirmed the so-called obesity paradox, whereby possibly because adipose tissue appears as an active participant in regulating physiological and pathological processes in hypermetabolic states, adipocytes can not only secrete various hormones and bioactive peptides but also produce and release pro- and anti-inflammatory factors for immune regulation. This may attenuate inflammatory responses during acute disease and improve survival (Fantuzzi, 2005; Torres et al., 2019).

This study explored the relationship between duration of antibiotic use and VAP and performed subgroup analyses to identify a cutoff of approximately 12 days for the prediction curve of antibiotic use duration and quantified the risk across the curve, and also found that obesity was associated with reduced ICU mortality in patients with VAP. Future research should explore other approaches for determining the optimal duration of antibiotic use to help clinicians make better decisions for patients with VAP in ICUs.

### Limitations

This study had some limitations. First, the data for this study originated in the MIMIC database. There was a potential risk of bias because most participants in this database were white Americans, which restricts the generalizability of the conclusions. Second, some patient indicators were not completely reported, which resulted in information leakage. Third, the severity of VAP could not be precisely determined. Fourth, the retrospective design of the study meant that some other biases were undoubtedly present. Fifth, patient functional outcomes and post-discharge disposition for ventilator-associated pneumonia were unknown due to a lack of long-term follow-up procedures in the MIMIC-IV database. Despite these limitations, we believe that determining the optimal antibiotic duration can help to better understand the prognosis of patients with VAP in ICUs.

## Conclusions

Considering that patients with VAP are more likely to have unfavorable clinical outcomes, selecting the appropriate antibiotic use duration is critical. VAP patients have a certain risk of death in the ICU, and our study found that the duration of antibiotic use had a nonlinear effect on the prognosis of VAP patients, with the lowest risk of in-hospital mortality in VAP when the duration of antibiotic use was 12 days, which may be a turning point in the prognosis of VAP patients. And so an accurate and reliable prognostic model for the best antibiotic duration to forecast VAP still needs to be developed.

In the furture, we will to keep researching the impact of antibiotic use duration on VAP. In order to develop a predictive model with accurate thresholds, sensitivity, and selectivity, as well as a high level of reliability, to help clinicians make concise management and treatment decisions for the prognosis of and maximizing the survival chances of patients with VAP.

## Data Availability

The raw data supporting the conclusions of this article will be made available by the authors, without undue reservation.

## Abbreviations

APSIII, acute physiology score-III; ALT, alanine aminotransferase; BMI, body mass index; CIMV, continuous invasive mechanical ventilation for less than 96 consecutive hours; CKD, chronic kidney disease; CCI, charlson comorbidity index; CI, confidence interval; DBP, diastolic blood pressure; GC, gastric catheterization; GCS, Glasgow Coma Scale; HR, hazard ratio; ICU, intensive care unit; LODS, logistic organ dysfunction score; MELD, model for end-stage liver; MIMIC-IV, medical information mart for intensive care IV; MIC, minimum inhibitory concentration; MBP, mean blood pressure; PEG, percutaneous (endoscopic) gastrostomy; POUF, performance of urinary filtration; PK/PD, pharmacokinetics and pharmacodynamics; RV, respiratory ventilation for more than 96 consecutive hours; RCS, restricted cubic spline; RBC, red blood cell; RDW, red blood cell distribution width; SCR, serum creatinine; SOFA, sequential organ failure assessment; SAPSII, simplified acute physiology score-II; TT, temporary tracheostomy; VAP, ventilator-associated pneumonia.
